# How Duffy Blood Group (*FY*) Polymorphism and Age Modulate Vivax Malaria Risk at the Community Level: A Population-based Retrospective Cohort Study in the Amazon

**DOI:** 10.1093/infdis/jiaf562

**Published:** 2025-11-06

**Authors:** Carlos A Prete, Taís N de Sousa, Isabela M Naziazeno, Maria Carolina S B Puça, Winni A Ladeia, Priscila T Rodrigues, Igor C Johansen, Gilberto A Paula, Marcelo U Ferreira, Rodrigo M Corder, the Mâncio Lima Cohort Study Working Group, Alexandre S Nogueira, Anderson R J Fernandes, Andreea-Beatrice Rusu, Bárbara Prado C Silva, Igor C Johansen, Isabel Giacomini, Jaques N de Carvalho, Juliana C Belizário, Juliana Tonini, Lais C Salla, Marcelo U Ferreira, Maria José Menezes, Pablo S Fontoura, Priscila R Calil, Priscila T Rodrigues, Rodrigo M Corder, Thaís C de Oliveira, Vanessa C Nicolete, Winni A Ladeia, Amanda O S Fernandes, Rodrigo M Martorano, Paulo E M Ribolla, Simone Ladeia-Andrade, Carlos E Cavasini, Joseph M Vinetz, Marcia C Castro

**Affiliations:** Department of Parasitology, Institute of Biomedical Sciences, University of São Paulo, São Paulo, Brazil; Department of Communications, School of Electrical and Computer Engineering, State University of Campinas, Campinas, Brazil; Molecular Biology and Malaria Immunology Research Group, René Rachou Institute, Fiocruz, Belo Horizonte, Brazil; Department of Microbiology, Tumor and Cell Biology, Karolinska Institutet, Solna, Sweden; Molecular Biology and Malaria Immunology Research Group, René Rachou Institute, Fiocruz, Belo Horizonte, Brazil; Molecular Biology and Malaria Immunology Research Group, René Rachou Institute, Fiocruz, Belo Horizonte, Brazil; Department of Parasitology, Institute of Biomedical Sciences, University of São Paulo, São Paulo, Brazil; Department of Parasitology, Institute of Biomedical Sciences, University of São Paulo, São Paulo, Brazil; Brazilian Center for Research in Energy and Materials, Campinas, Brazil; Department of Parasitology, Institute of Biomedical Sciences, University of São Paulo, São Paulo, Brazil; Department of Demography, Institute of Philosophy and Humanities, State University of Campinas, Campinas, Brazil; Department of Statistics, Institute of Mathematics and Statistics, University of São Paulo, São Paulo, Brazil; Department of Parasitology, Institute of Biomedical Sciences, University of São Paulo, São Paulo, Brazil; Global Health and Tropical Medicine (GHTM), Associate Laboratory in Translation and Innovation Towards Global Health (LA-REAL), Institute of Hygiene and Tropical Medicine, NOVA University of Lisbon, Lisbon, Portugal; Department of Parasitology, Institute of Biomedical Sciences, University of São Paulo, São Paulo, Brazil; Global Health and Tropical Medicine (GHTM), Associate Laboratory in Translation and Innovation Towards Global Health (LA-REAL), Institute of Hygiene and Tropical Medicine, NOVA University of Lisbon, Lisbon, Portugal; Department of Life Sciences, Faculty of Natural Sciences, Imperial College London, London, United Kingdom

**Keywords:** malaria, *plasmodium vivax*, duffy blood group, *FY* polymorphism, zero-inflated negative binomial

## Abstract

**Background:**

A promoter variant commonly found in sub-Saharan Africans and their descendants disrupts Duffy antigen (Fy) expression on erythrocytes, leading to the Fy-negative phenotype, and confers partial resistance to blood-stage *Plasmodium vivax* infection. In addition, the 125G→A substitution, rare in Africans, defines the Fy^a^/Fy^b^ polymorphism that can modulate vivax malaria risk in Amazonians. The combined effect of these *FY* polymorphisms on *P. vivax* infection risk remains little explored at the population level.

**Methods:**

We studied a household-based random sample of 1737 Amazonians, with a well-balanced distribution of *FY* alleles, who were exposed to *P. vivax* transmission and contributed 7878.9 person-years of follow-up. We fitted a multivariable zero-inflated negative binomial model to incidence data, assuming that zero counts could arise from individuals at risk who remained uninfected over 5 years of follow-up (“sampling zeroes”) or from not-at-risk individuals (“structural zeroes”).

**Results:**

*Plasmodium vivax* infections were heterogeneously distributed in the population, with 0 to 11 cases per person (average incidence, 25.8 cases/100 person-years at risk). We show that Fy-negativity remains a major malaria resistance trait in Amazonians and contributes significantly to the “structural zeroes” observed in *P. vivax* incidence data. Moreover, the differences in *P. vivax* infection risk associated with the Fy^a^/Fy^b^ polymorphism observed among young participants were attenuated with increasing age, most likely because more susceptible Fy(b+) individuals develop clinical immunity faster than less susceptible Fy(a+) individuals.

**Conclusions:**

*FY* polymorphism appears to modulate the rate at which immunity to *P. vivax* develops in Amazonians, with clear clinical and public health implications.

## Teaser

Duffy (Fy) negativity in admixed Amazonians confers strong resistance to *Plasmodium vivax* infection, while the Fy^a^/Fy^b^ polymorphism among Fy-positives appears to modulate the rate at which age-dependent immunity to vivax malaria develops, with clear clinical and public health implications.

Malaria transmission persists in the Americas, with 505 600 locally acquired cases (73% caused by *Plasmodium vivax*) recorded in 2023 and an estimated 139 million people at risk of infection living in 16 countries and one territory. The Amazon Basin is the main endemic area that accounts for more than 90% of the malaria cases on the continent [1].

The burden of *P. falciparum* malaria is heterogeneously distributed within communities, with 20% of the individuals experiencing nearly 80% of the infections, with clear implications for control and elimination efforts [2]. How the individual risk of malaria varies in areas where *P. vivax* predominates has been little studied, but the 20/80 rule appears to apply to vivax malaria in the Amazon: few individuals are repeatedly infected while most remain uninfected for long periods, leading to a zero inflation in population-wide incidence data due to either incompletely characterized inherited factors, acquired immunity, behavior, or merely by chance [3]. Mathematical model simulations indicate that the 20% of the people contribute 86% of the vivax malaria cases in the community [4].

The present analysis focuses on polymorphism in the Duffy blood group antigen (Fy or CD234) that modulates the susceptibility to *P. vivax* infection [5]. The entry of *P. vivax* blood stages into host cells—immature red blood cells (RBCs) known as reticulocytes—requires sequential interactions of ligand proteins on extracellular merozoites with receptors on the reticulocyte surface [6]. One key receptor-ligand interaction involves the region II of the Duffy-binding protein (PvDBP), expressed by *P. vivax* merozoites, that recognizes the Fy antigen expressed on the RBC surface [7]. Of note, high levels of naturally acquired IgG antibodies that block the interaction between PvDBP and the Fy receptor are associated with a decreased risk of vivax malaria in Amazonians [8].

Most sub-Saharan Africans and their descendants are homozygous for a *FY* gene promoter variant that disrupts Duffy antigen expression on RBCs, leading to the Fy-negative phenotype defined by serology [5]. Fy-negative people are at least partially resistant to *P. vivax* infection, but fully susceptible to *P. falciparum* [9, 10]. Approximately 3–6% of the Amazonians are Fy-negative [11, 12], but genotypically Fy-negatives experience occasional vivax malaria episodes [13], most likely because the parasite can invade a subset of erythroid precursors that transiently express Fy [14, 15]. *Plasmodium vivax* entry into RBCs can also be reduced by the common G to A substitution at nucleotide 125 of the *FY* gene coding sequence, which defines the Fy(a+) phenotype, with reduced affinity for the PvDBP ligand [5]. Fy(a+) Amazonians appear to have a significantly reduced risk of *P. vivax* infection [11, 16].

Here, we examine the contribution of *FY* polymorphism to the heterogeneity in *P. vivax* infection risk in Amazonians. We show that Fy-negatives are rarely infected with *P. vivax* and contribute significantly to the excess of zeroes in incidence density data. Moreover, vivax malaria incidence varies markedly with age, which in turn modifies the effect of *FY* genotype on infection risk, most likely because more susceptible people eventually develop clinical immunity after repeated infections. These complex interactions should be considered in studies of clinical immunity to *P. vivax* in Amazonians.

## METHODS

### Study Area and Population

Mâncio Lima (7°36′28.6″ S, 72°54′23.0″ W), in the Juruá Valley region of Acre State (Supplementary Figure 1), is the main urban malaria hotspot of Brazil. The site is characterized by an equatorial humid climate, with heavy rains between November and April and year-long malaria transmission [17]. The primary vector, *Anopheles* (*Nyssorhynchus*) *darlingi*, thrives in numerous fish farming tanks that were opened in the region starting in the mid-2000s [18].

A population census between November 2015 and April 2016 enumerated 9124 residents in the town of Mâncio Lima, distributed into 2329 households [17]. In April 2018, we randomly drawn approximately 20% of the households from the census listings and invited their residents to participate in an open cohort study to investigate biological and sociodemographic drivers of malaria transmission [19]. Capillary blood samples were obtained from 2774 participants during cross-sectional surveys carried out until November 2021 [20]. A total of 1921 study participants (69.2%) present in at least two cross-sectional surveys had their *FY* genotype determined.

### Retrospective Cohort and Malaria Case Records

**Figure 1.**
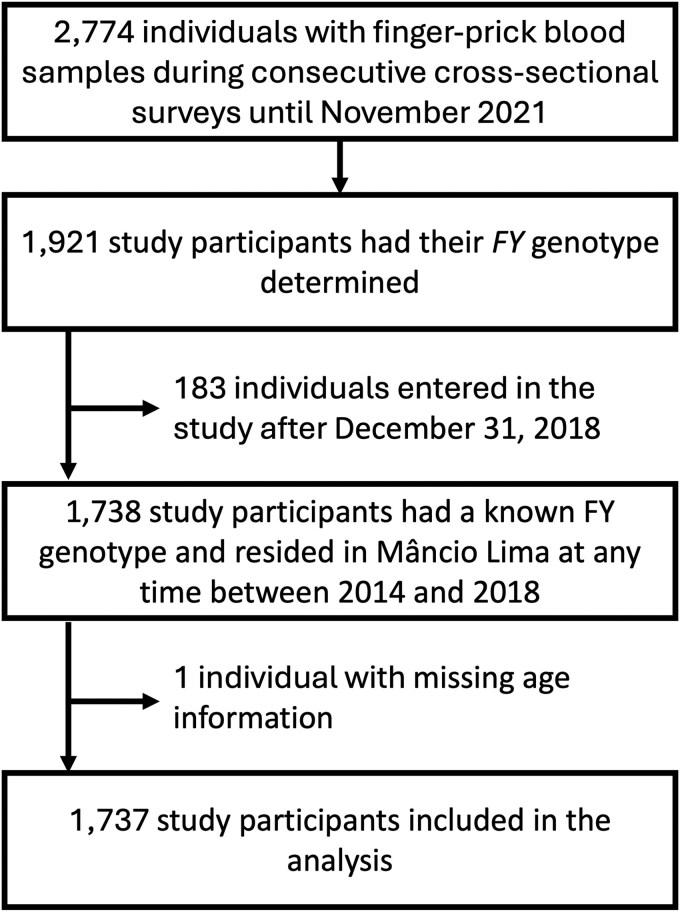
Study flow diagram. Finger-prick blood samples were collected from 2774 participants between April 2018 and November 2021 and the *FY* genotype was determined for 1921 participants. Of those, one participant was excluded due to missing age information, and 183 were excluded because they did not reside in the study site anytime between 01 January 2014 and 31 December 2018, resulting in 1737 study participants included in the retrospective cohort.

Figure 1 summarizes the steps for retrospective cohort construction. Eligible participants were residents in Mâncio Lima anytime between 2014 and 2018 who had known *FY* genotype. The date of entry in the retrospective cohort was defined as the date of birth, the date the participant moved into his/her residence in Mâncio Lima, for those not enumerated in the 2015–2016 census, or 1 January 2014—whatever was the most recent. The dates of entry and exit were considered to estimate the number of person-years at risk; participants who left the study area before 31 December 2018 were considered lost to follow-up since the date they moved away (Supplementary Methods online).

The primary study outcome is single-species *P. vivax* infection, irrespective of parasite density and presence of symptoms, diagnosed by thick-smear microscopy or rapid diagnostic test among cohort participants from 1 January 2014 through 31 December 2018. We retrieved from the electronic notification database (SIVEP-Malaria) of the Ministry of Health of Brazil [21] malaria cases notified during the study period that matched participants′ name, sex, age, and date of birth, in addition to their mothers′ name (Supplementary Methods online). This strategy retrieved virtually all malaria cases in cohort participants, since malaria is a notifiable disease in Brazil and diagnostic testing (required for treatment) and antimalarial medications are freely and widely available only within the public sector. An interval equal or > 28 days between two or more consecutive episodes was required to count the latter episode as a new malaria infection; when different species were detected < 28 days apart, the participant was considered to have a single episode of mixed-species infection (Supplementary Figure 2).

### Laboratory Analysis

We genotyped two single-nucleotide polymorphisms at the *FY* locus (also known as *Atypical Chemokine Receptor 1* or *ACKR1* gene): (i) The −67T→C substitution in the RBC-specific globin transcription factor-1 (GATA-1) binding motif (rs2814778), which suppresses Fy expression on the erythrocyte surface; FY*01N.01 allele homozygosity leads to the Fy(a−b−) or Fy-negative (null) phenotype, while heterozygotes for the FY*01N.01 allele have their Fy erythrocyte surface expression reduced by 50%. (ii) The 125G→A substitution in exon 2 (rs12075), which differentiates the FY*01 and FY*02 alleles associated with the Fy(a+) and Fy(b+) phenotypes, respectively (Table 1).

**Table 1.** Duffy Blood Group (*FY*) Genotypes and Phenotypes Characterized in This Study

*FY* Genotype	Duffy Blood Group Phenotype	Fy Expression Levels On Red Blood Cells
FY*01/FY*01	Fy(a+)	2 × Fy (a+), 0 × Fy (b+)
FY*01/FY*01N.01	Fy(a+)	1 × Fy (a+), 0 × Fy (b+)
FY*02/FY*02	Fy(b+)	2 × Fy (b+), 0 × Fy (a+)
FY*02/FY*01N.01	Fy(b+)	1 × Fy (b+), 0 × Fy (a+)
FY*01/FY*02	Fy(a + b+)	1 × Fy (a+), 1 × Fy (b+)
FY*01N.01/FY*01N.01	Fy(a− b−)	0 × Fy (a+), 0 × Fy (b+)

Nomenclature according to the International Society of Blood Transfusion (https://www.isbtweb.org/resource/008fy.html) and Fy expression data according to Zimmerman *et al*. (2013) [5].

Template DNA was extracted from 50-µL blood samples using DNA Investigator kits (Qiagen, Hilden, Germany). We used TaqMan assays (C__15769614_10 and C 2493442_20), with hydrolysis probes labeled with the VIC and FAM fluorochromes (ThermoFisher Scientific, Waltham, MA) [22]. The PCR amplification was carried out on a ViiA7 Real-Time PCR System (ThermoFisher Scientific) essentially as described [22], with one cycle of polymerase activation at 95°C for 10 minutes, followed by 50 cycles of denaturation at 95°C for 15 seconds and annealing/extension at 60°C for 1 minute (Supplementary Methods).

### Statistical Analysis

We explored the association between *FY* genotype and the risk of *P. vivax* infection while adjusting for potential confounders, namely sex, age (0–16, 17–40, and >40 years), and wealth index terciles, a proxy of socioeconomic status [20]. We fitted distributional zero-inflated negative binomial (ZINB) regression models [23] to individual-level data using the *R* package *gamlss* [24]. The ZINB model combines the negative binomial (NB) and a binary Bernoulli distribution to fit overdispersed incidence data with zero inflation—ie, with more zeroes than expected under a conventional NB. A zero outcome may originate from two different processes: some people are at risk, but remain uninfected (“sampling zeroes”), while others are intrinsically free of malaria risk (“structural zeroes”) during the study (Supplementary Figure 4). At-risk individuals contribute vivax malaria cases according to the NB function while not-at-risk individuals necessarily contribute zero cases and account for the zero inflation in the data set [17]. We obtained (i) incidence rate ratio (IRR) estimates, along with 95% confidence intervals (CIs), to quantify the influence of each covariate on vivax malaria incidence, while controlling for all other covariates in the NB model, and (ii) odds ratios (ORs), along with 95% CIs, to identify individual characteristics that affect the odds of belonging to the “structural zeroes” component of the logit model. Because malaria incidence varied over time and participants differed in follow-up duration, a covariate that represents individual's time at risk weighted by daily malaria incidence was added to the NB and binary components of the model [25].

To select the final model, we fitted candidate models to a training data set comprising 70% of the data and applied the models to a test data set comprising the remaining 30% of the data. We used the *gamlssVGD* function [26] to compare the predictive performance of candidate models and selected the model with the smallest global deviation in the test set. The selected model was subsequently refitted to the entire dataset. To examine the distribution of its residuals, we used a normal quantile-quantile (Q-Q) plot and a randomized detrended normal Q-Q plot, also known as worm plot [27].

### Ethical Approval and Consent to Participate

The Institutional Review Board of the Institute of Biomedical Sciences, University of São Paulo, and by the National Committee of Ethics in Research of the Ministry of Health of Brazil (CAAE number 64767416.6.0000.5467) approved the study protocol. Written informed consent and assent were obtained from all study participants or their parents/guardians.

## RESULTS

We studied 1737 people with known *FY* genotype distributed into 620 households, who were aged between <1 and 104 years (mean, 29.1 years; median, 25.1) and contributed 7878.9 person-years of follow-up (Figure 1). Between 1 January 2014 and 31 December 2018, they experienced 2033 infections with *P. vivax,* 298 with *P. falciparum,* and 38 co-infections with both species (Supplementary Figure 2), mostly (2325 or 98.1%) diagnosed through passive case detection among symptomatic patients attending local malaria clinics. Mixed-species infections were not considered in the main analysis because reported symptoms could be due to the presence of *P. falciparum*, *P. vivax*, or both species. We estimated the average incidence of *P. vivax* infection at 25.8 cases/100 person-years at risk. Cases were heterogeneously distributed in the population, with a mean of 1.2 (range, 0 to 11 and variance of 3.0) *P. vivax* infections per person over five years. While 930 (53.5%) participants remained free of vivax malaria during the follow-up, 317 (18.2%) had three or more infections each and together accounted for 1351 cases, nearly two thirds of the total (Supplementary Figure 4).

### *Plasmodium vivax*: Rare but Not Absent in Fy-Negative Amazonians

Table 2 shows malaria incidence rates according to *FY* genotype. Fy-negatives were not completely resistant to blood-stage *P. vivax* infection; to the contrary, 15 Fy-negative participants aged between 3 and 49 years experienced 19 microscopy-confirmed infections, all of them symptomatic (Supplementary Table 1), with an incidence density of 4.0/100 person-years at risk (6 to 8-fold lower than that in Fy-positives). This is the largest series of vivax malaria cases among Fy-negative Amazonians. The proportion of FY*01 N.01/FY*01N.01 homozygotes significantly exceeded the Hardy-Weinberg expectation, consistent with some level of inbreeding in the study population (Supplementary Table 2).

**Table 2.** Malaria Incidence Density According to Duffy Blood Group (*FY*) Genotype in the Study Population of Mâncio Lima, Brazil, 2014–2018

			*Plasmodium Vivax*		*Plasmodium Falciparum*	
*FY* genotype	No. (%) of participants	Person-years	No. of cases	Incidence (95% CI)	No. of cases	Incidence (95% CI)
FY*01/FY*01	251 (14.4%)	1137.50	298	26.2 (23.3–29.3)	45	4.0 (2.9–5.3)
FY*01/FY*01N.01	285 (16.4%)	1324.60	331	25.0 (22.4–27.8)	57	4.3 (3.3–5.6)
FY*02/FY*02	285 (16.4%)	1277.40	392	30.7 (27.7–33.9)	48	3.8 (2.8–5.0)
FY*02/FY*01N.01	279 (16.1%)	1254.60	314	25.0 (22.3–28.0)	50	4.0 (3.0–5.3)
FY*01/FY*02	531 (30.6%)	2406.60	679	28.2 (26.1–30.4)	80	3.3 (2.6–4.1)
FY*01N.01/FY*01N.01	106 (6.1%)	478.20	19	4.0 (2.4–6.2)	18	3.8 (2.2–5.9)
Total	1737 (100.0%)	7878.80	2033	25.8 (24.7–26.9)	298	3.8 (3.4–4.2)

Incidence per 100 person-years at risk; CI, confidence interval.

### Fy-Negativity and Zero Inflation in *P. vivax* Incidence Data

The average probability parameter of the binary component of the ZINB model was 47.5% (95% CI, 47.3–47.7%); this means that approximately 825 study participants were classified as “structural zeroes” accounting for the excess of zero counts beyond the NB expectations, after adjusting for all covariates (Supplementary Figure 4A). We found that Fy-negatives contributed significantly to the excess of zeroes, with an odds ratio of 7.32 (*P* < .001), compared with Fy-positives, of being an “structural zero” free of any vivax malaria risk (Table 3). Participants aged between 17 and 40 years—the age group with the highest average malaria incidence—had a significantly smaller odds of being a “structural zero”, compared with the reference 0–16 years group (Table 3).

**Table 3.** Factors Associated With Vivax Malaria Incidence Density Identified by Multivariable Zero-inflated Negative Binomial (ZINB) Regression Analysis in the Study Population of Mâncio Lima, Brazil, 2014–2018

	Count Component (Negative Binomial Mean)			
Variable	No.	IRR	95% CI	*P*
Age	…	…	**…**	
0–16 y	558	1	Reference	
17–40 y	723	1.226	0.860–1.747	.261
> 40 y	456	1.075	0.700–1.652	.741
Sex	**…**	**…**	**…**	**…**
Female	895	1.00	Reference	
Male	842	1.066	0.952–1.192	.268
Wealth index	…	**…**	**…**	**…**
Lowest	596	1.00	Reference	
Intermediate	581	0.935	0.818–1.069	.324
Highest	560	0.842	0.729–0.973	.020
*FY* genotype (for age 0–16 y)	**…**	**…**	**…**	**…**
FY*01/FY*01N.01	94	1.00	Reference	
FY*01/FY*01	78	0.899	0.578–1.400	.639
FY*02/FY*01N.01	87	1.568	1.061–2.316	.024
FY*02/FY*02	90	1.296	0.860–1.952	.216
FY*01/FY*02	173	1.220	0.861–1.729	.264
FY*01N.01/FY*01N.01	36	0.402	0.086–1.874	.246
Interaction age: *FY* genotype	…	**…**	**…**	**…**
Age 17–40 : FY*01/FY*01N.01	104	-	-	
Age 17–40 : FY*01/FY*01	120	1.303	0.779–2.181	.313
Age 17–40 : FY*02/FY*01N.01	126	0.542	0.335–0.877	.013
Age 17–40 : FY*02/FY*02	117	0.895	0.559–1.434	.646
Age 17–40 : FY*01/FY*02	209	0.827	0.546–1.252	.370
Age 17–40 : FY*01N.01/FY*01N.01	47	1.197	0.235–6.095	.829
Age >40 : FY*01/FY*01N.01	69	-	-	
Age >40 : FY*01/FY*01	71	1.028	0.560–1.886	.930
Age >40 : FY*02/FY*01N.01	69	0.625	0.356–1.100	.103
Age >40 : FY*02/FY*02	75	0.847	0.469—1.530	.583
Age >40 : FY*01/FY*02	149	0.934	0.568–1.535	.788
Age >40 : FY*01N.01/FY*01N.01	23	1.923	0.301–12.278	.490
Time at risk, weighted by incidence (log)		1.490	1.125–1.974	.005
Intercept		1.961	1.429–2.691	<.0001

	Binary Component (Logistic)			
Variable	No.	OR	95% CI	P
Age	…	**…**	**…**	**…**
0–16 y	558	1.00	Reference	
17–40 y	723	0.473	0.350–0.640	<.0001
> 40 y	456	0.940	0.682–1.297	.708
Fy phenotype deduced from genotype	…	**…**	**…**	**…**
Fy-positive	1631	1.00	Reference	**…**
Fy-negative	106	7.317	3.331–16.070	<.0001
Time at risk, weighted by incidence (log)		0.349	0.231–0.526	<.0001
Intercept		0.971	0.774–1.220	.803

	Negative Binomial Dispersion Parameter *k*			
Intercept		0.150	0.088–0.255	<.0001

IRR, incidence rate ratio; OR, odds ratio.

### *FY* Genotypes and Vivax Malaria Risk: Age as an Effect Modifier

**Figure 2.**
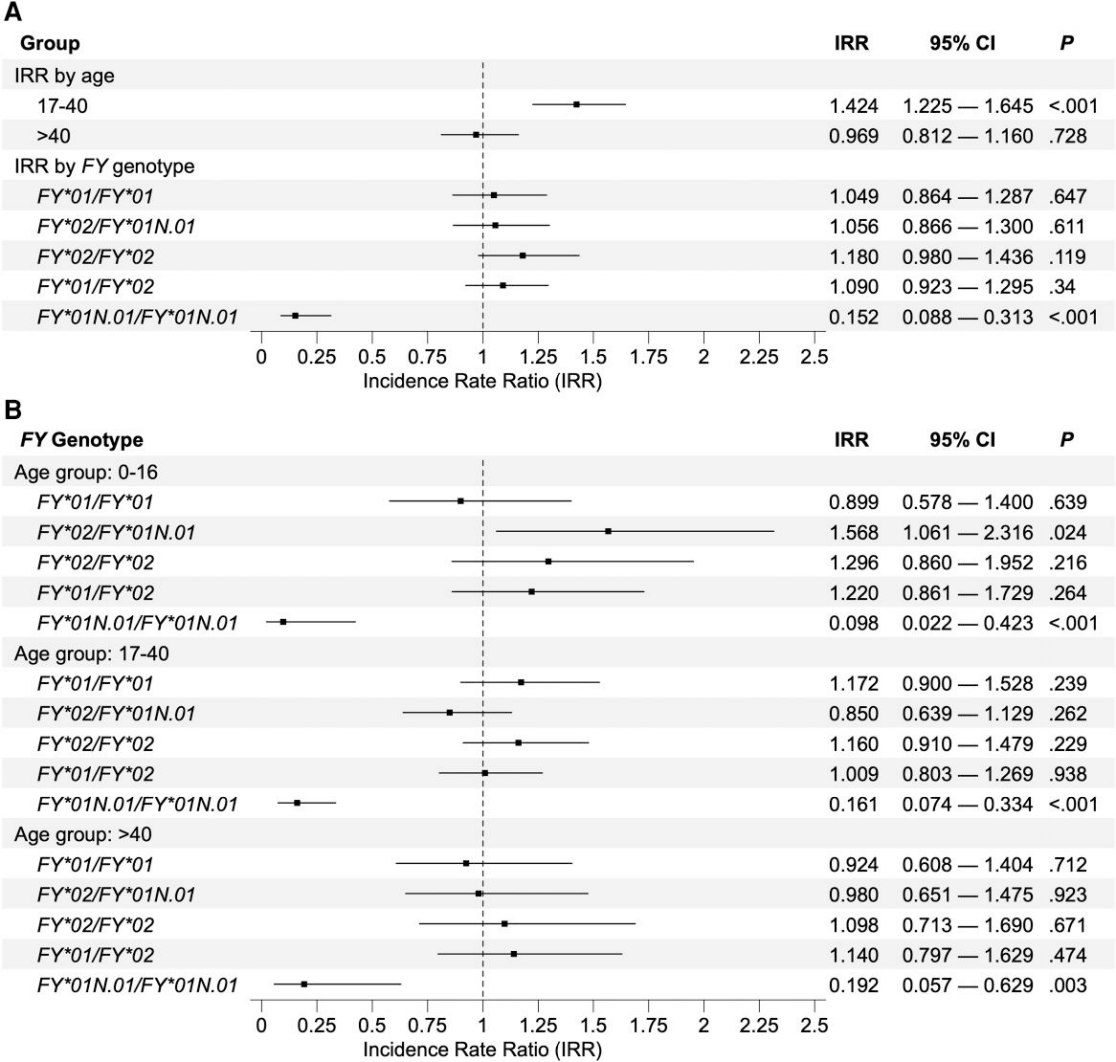
Incidence rate ratios (IRRs) of infection with *Plasmodium vivax* across *FY* genotypes in the study population of Mâncio Lima, Brazil, between 2014 and 2018. *A*, All age groups combined; (*B*) IRRs stratified by age group (0–16, 17–40 and >40 y). Estimates were adjusted for age (only panel *A*), sex, wealth index tercile, and log-transformed time at risk weighted by malaria incidence and for an interaction between age and *FY* genotype with multivariable zero-inflated negative binomial regression analysis (Table 3).

Similar incidence densities of *P. vivax* infection, ranging between 25.0 and 30.7 cases/100 person-years at risk, were found in all Fy-positives, regardless of the *FY* genotype (Table 2). The multivariable ZINB model identified greater wealth as a correlate of decreased *P. vivax* incidence, but failed to detect significant differences in IRRs across Fy-positive genotypes, using FY*01/FY*01N.01 as the reference, after adjusting for age, sex, wealth index tercile, and log-transformed time at risk weighted by malaria incidence (Figure 2*A*). The largest IRR was associated with the FY*02/FY*02 genotype, although not significantly. This result does not suggest that, in this population, the 125G→A nucleotide substitution defining the Fy^a^/Fy^b^ polymorphism affects significantly the risk of vivax malaria among Fy-positives.

**Figure 3.**
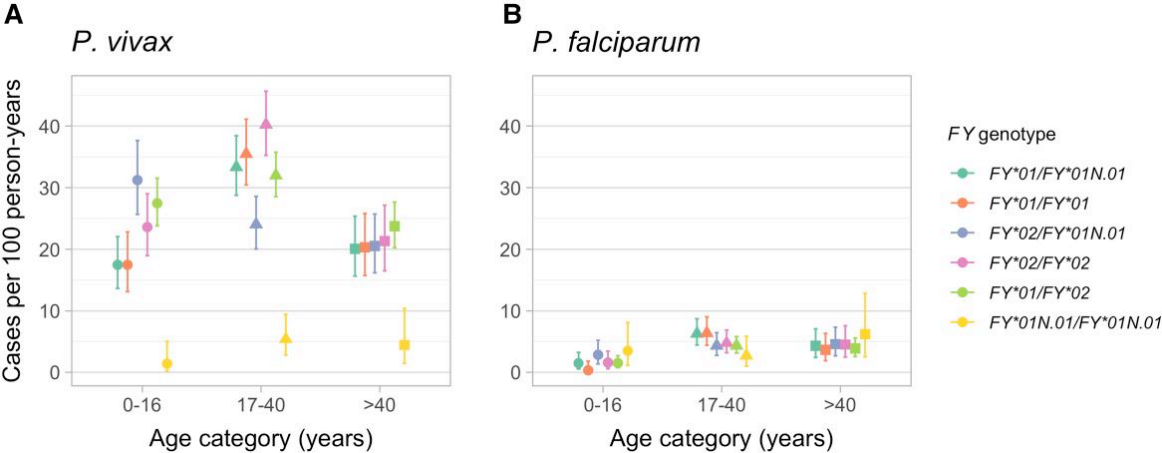
Incidence density of *Plasmodium vivax* (*A*) and *P. falciparum* (*B*) infections in Mâncio Lima, Brazil, 2014–2018, by age group (0–16, 17–40, > 40 y) and *FY* genotype (colors). Symbols denote mean values (circles, 0–16; triangles, 17–40; squares, > 40); bars indicate 95% confidence intervals. *FY* genotypes are defined in Table 1.

Nevertheless, *P. vivax* infection incidence varied markedly with age and, more importantly, the magnitude of the association between the Fy^a^/Fy^b^ polymorphism and vivax malaria incidence differed by age (Figure 3*A*). In the 0–16 years stratum, Fy(b+) participants carrying the FY*02/FY*01N.01 genotype had the highest risk of *P. vivax* infection, while Fy(a+) participants carrying the FY*01/FY*01N.01 genotype had the lowest infection incidence among Fy-positives, consistent with results from a previous study of Amazonians [11]. By contrast, the FY*02/FY*01N.01 genotype was associated with a lower incidence among participants aged 17–40 years, compared with those aged 0–16 years. The oldest (>40 years) age group was characterized by quite similar *P. vivax* incidence rates across all Fy-positive genotypes. Accordingly, the vivax malaria risk estimated by the multivariable ZINB model was significantly higher in the 17–40 years strata, compared with younger study participants (Figure 2*A*). The incidence of *P. falciparum* infection—average, 3.8 cases/100 person-years at risk—remained low across all age groups and *FY* genotypes (Figure 3*B*).

We found evidence that age might modify the protective effect from *P. vivax* infection associated with the Fy^a^/Fy^b^ polymorphism. To explore this hypothesis, we first tested for interactions between age and *FY* genotype using the NB component of the multivariable ZINB model. We found that the impact of the FY*02/FY*01N.01 genotype on infection risk was attenuated by 46% among adults aged 17–40 years (IRR = 0.54; *P* = .013), compared with children and adolescents aged 0–16 years (Table 3).

Next, we examined how *P. vivax* incidence rate ratios varied across *FY* genotypes within age strata. Children and adolescents aged 0–16 years who carried the FY*02/FY*01N.01 genotype had a 57% greater incidence, compared with their counterparts carrying the low-risk FY*01/FY*01N.01 genotype, after adjusting for sex, wealth index tercile, and time at risk (Figure 2*B*). By contrast, no difference was found among older participants, with relatively similar incidence rates of *P. vivax* infection between the FY*02/FY*01N.01 and FY*01/FY*01N.01 genotypes. Fy-negativity was associated with a greatly reduced IRR of vivax malaria across all age groups. We conclude that increasing age attenuates the positive association between the FY*02/FY*01N.01 genotype and *P. vivax* infection risk seen in the youngest age group (Figure 2*B*).

## DISCUSSION

Fy-negativity offers a textbook example of adaptive evolution in malaria-exposed populations from sub-Saharan Africa, where the FY*01N.01 allele is at near fixation and *P. vivax* infections are typically infrequent. However, the interplay between Fy and vivax malaria in the Brazilian Amazon is complicated by the high frequencies of the three major *FY* alleles in local populations [11, 12, 16, 28], with interesting clinical and public health implications.

Data from our cohort study show that *FY* polymorphism modulates population-level vivax malaria risk in different ways. First, and not surprisingly, FY*01N.01 allele homozygosity is associated with a very low risk of infection among Amazonians of all age groups. *Plasmodium vivax* infections do occur in Fy-negative Amazonians (Supplementary Table 1), but much less frequently than in some sub-Saharan African settings [10], such as Madagascar [29]. Indeed, Fy-negatives (6% of the participants) contributed significantly to the excess of zeroes seen in vivax malaria incidence data, suggesting that most of them would remain uninfected even in the absence of interventions such as chemoprophylaxis, immunization, or bed net distribution. The zero-inflated component in our statistical model effectively captures this subgroup, improving estimation accuracy. As argued elsewhere, the effect of interventions for malaria control and elimination may be severely underestimated if deployed to populations with large proportions of structural zeroes [30].

Second, vivax malaria risk among Fy-positive Amazonians is modulated by a previously undescribed interaction between age and Fy^a^/Fy^b^ polymorphism. Consistent with the stronger binding affinity between PvDBP and the Fy^b^ antigen, compared with Fy^a^ [16], young Fy(b+) participants carrying the FY*02/FY*01N.01 genotype were more frequently infected with *P. vivax* than their Fy(a+) counterparts carrying the FY*01/FY*01N.01 genotype, but this risk difference was attenuated with increasing age and disappeared among older adults (Figure 2*B*). This finding is supported by our regression model that jointly evaluates the effects of age, *FY* genotype, and their interaction on incidence, while adjusting for other covariates known to influence *P. vivax* infection risk.

Adaptive immunity to *P. vivax* blood stages gradually develops after repeated infections and is largely antibody-mediated [31]. Naturally acquired antibodies can inhibit the PvDBP-Fy interaction [8, 32], partially blocking parasite entry into RBCs [32, 33] and reducing the risk of blood-stage *P. vivax* infection [34] and clinical malaria [8]. Antibody-dependent protection from vivax malaria is arguably more efficient in Fy(a+) individuals, since anti-PvDBP antibodies are more likely to block the lower-affinity binding of parasites to Fy(a+) RBCs compared with Fy(b+) RBCs [16]. PvAMA-1 and several merozoite surface antigens are additional major blood-stage targets of naturally acquired immunity to *P. vivax* [31].

Our results add an extra layer of complexity to the association between Fy and immunity in *P. vivax* infection. They suggest that Fy(b+) people, who are initially more susceptible to *P. vivax* and more likely to be repeatedly infected throughout childhood and adolescence, become partially immune to malaria faster than their less susceptible Fy(a+) counterparts. As a result, the impact of the Fy^a^/Fy^b^ polymorphism on *P. vivax* infection risk may be concealed in populations exposed to intense malaria transmission, such as that in our study (Figure 2*A*), in the absence of age-stratified data analysis (Figure 2*B*). Likewise, acquired immunity among immigrants has been suggested to equalize vivax malaria risk across *FY* genotypes after decades of residence in the Amazon [11]. We hypothesize that the clinical immunity acquired over time might eventually counterbalance the greater susceptibility to vivax malaria in Fy(b+) individuals. When exposed to intense malaria transmission since birth, high-risk children experience repeated *P. vivax* infections and become partially immune adults who constitute an asymptomatic reservoir of parasites that fuels onward transmission [4]. By contrast, individuals exposed to low transmission are less likely to experience enough infections to develop a strong clinical immunity and baseline differences in susceptibility to vivax malaria associated with the Fy^a^/Fy^b^ polymorphism may persist in older age groups (eg [16]).

Two aspects make the Mâncio Lima Cohort Study ideally suited for our analyses: (i) this is the largest population-based study ever carried out to address the association between *FY* genotypes and vivax malaria in the Amazon and (ii) the highly admixed study population [35] has a well-balanced distribution of *FY* alleles and was exposed to intense *P. vivax* transmission between 2014 and 2018 (Table 2). However, this study has some limitations. First, malaria case records were retrieved retrospectively from a passive surveillance database and no blood samples were available for further confirmatory diagnostic testing. We missed most transient or chronic submicroscopic (often asymptomatic) infections that did not prompt a visit to a health facility. Therefore, our findings primarily reflect the epidemiology of symptomatic *P. vivax* infections. Second, analyses of passively detected malaria episodes may be prone to biases due to differences in access to health facilities. Although free testing and treatment for malaria were widely available in seven health posts distributed in Mâncio Lima, individual health-seeking behavior may vary among participants. Third, we may have missed relatively rare Fy phenotypes—such as Fy(b + ^w^)—that are not resolved by our genotyping strategy (https://www.isbtweb.org/resource/008fy.html). Finally, we assume that cohort participants gradually developed clinically immunity with increasing age, but did not measure laboratory correlates of immunity to malaria (eg, levels of biding-inhibitory anti-PvDBP IgG antibodies [8]) to test this hypothesis in the study population. Future studies are needed (i) to address the mechanisms underlying the gradual, age-dependent attenuation of vivax risk observed in individuals carrying *FY* genotypes associated with greater susceptibility to infection; and (ii) to elucidate why participants carrying the FY*02/FY*01N.01 genotype are at increased risk of *P. vivax* infection, compared with those with the FY*02/FY*02 genotype, despite having lower Fy(b+) expression levels on reticulocytes.

## CONCLUSION

We show that Fy-negativity remains a major malaria resistance trait in the Brazilian Amazon. In addition, the differences in *P. vivax* infection risk associated with the Fy^a^/Fy^b^ polymorphism are attenuated with age, most likely because more susceptible Fy(b+) individuals acquire partial immunity faster than less susceptible Fy(a+) individuals. We conclude that *FY* polymorphism—a key driver of community-wide heterogeneity in *P. vivax* infection risk—can modulate the rate at which clinical immunity develops and must be considered in epidemiological studies of vivax malaria in genetically admixed populations, such as those in the Brazilian Amazon.

## Supplementary Material

jiaf562_Supplementary_Data

## Supplementary Data

Supplementary materials are available at *The Journal of Infectious Diseases* online (http://jid.oxfordjournals.org/). Supplementary materials consist of data provided by the author that are published to benefit the reader. The posted materials are not copyedited. The contents of all supplementary data are the sole responsibility of the authors. Questions or messages regarding errors should be addressed to the author.
