# Supplementary material for: How Duffy Blood Group (FY) Polymorphism and Age Modulate Vivax Malaria Risk at the Community Level: A Population-based Retrospective Cohort Study in the Amazon
Source: J Infect Dis. 2025 Nov 6;233(3):e756–65. doi: 10.1093/infdis/jiaf562 (PMC13017048; doi:10.1093/infdis/jiaf562)
Supplement: jiaf562_Supplementary_Data [file jiaf562_supplementary_data.docx]

SUPPLEMENTARY DATA

How Duffy blood group polymorphism and age modulate vivax malaria risk at the community level: a population-based retrospective cohort study in the Amazon

Carlos A. Prete Jr.,^1,2^ Taís Nóbrega de Sousa,^3,4^ Isabela M. Naziazeno,^3^ Maria Carolina S. B. Puça,^3^ Winni A. Ladeia,^1^ Priscila T. Rodrigues,^1,5^ Igor C. Johansen,^1,6^, Gilberto A. Paula,^7^ Marcelo U. Ferreira,^1,8^ and Rodrigo M. Corder^1,8,9^ on behalf of the Mâncio Lima Cohort Study Working Group*

^1^Department of Parasitology, Institute of Biomedical Sciences, University of São Paulo, São Paulo, Brazil

^2^Department of Communications, School of Electrical and Computer Engineering, State University of Campinas, Campinas, Brazil

^3^Molecular Biology and Malaria Immunology Research Group, René Rachou Institute, Fiocruz, Belo Horizonte, Brazil

^4^Department of Microbiology, Tumor and Cell Biology, Karolinska Institutet, Solna, Sweden

^5^Brazilian Center for Research in Energy and Materials, Campinas, Brazil

^6^Department of Demography, Institute of Philosophy and Humanities, State University of Campinas, Campinas, Brazil

^7^Department of Statistics, Institute of Mathematics and Statistics, University of São Paulo, São Paulo, Brazil

^8^Global Health and Tropical Medicine (GHTM), Associate Laboratory in Translation and Innovation Towards Global Health (LA-REAL), Institute of Hygiene and Tropical Medicine, NOVA University of Lisbon, Lisbon, Portugal

^9^Department of Life Sciences, Faculty of Natural Sciences, Imperial College London, London, UK

**SUPPLEMENTARY METHODS**

**Study outcomes and record linkage strategy**

We considered all malaria infections, irrespective of parasite density and presence of symptoms, diagnosed by thick-smear microscopy or rapid diagnostic test among residents of Mâncio Lima between 1 January 2014 and 31 December 2018 (Supplementary Figure 2). Malaria case records were retrieved from the SIVEP-Malaria database, which included 47,447 infections diagnosed in residents of Mâncio Lima during the same period.

We first excluded records with “non-falciparum” results obtained with rapid diagnostic tests with the P.f/Pan format (*n* = 65). Such tests, which detect the histidine-rich protein 2 (HRP2) antigen of *P. falciparum* and a common, genus-specific lactate dehydrogenase (pLDH) of *Plasmodium* species, may miss falciparum malaria due to the high (46.5%) frequency of *HRP2* gene deletion in local *P. falciparum* populations [1]. Therefore, “non-falciparum” results may be obtained for infections with either *P. falciparum* (with HRP2 deletion) or *P. vivax* and were considered “undetermined species” (Supplementary Figure 2). However, results from rapid diagnostic tests with the P.f/P.v. format, which include separate bands with species-specific antibodies for the detection of pLDH from *P. falciparum* and *P. vivax,* were computed because *P. falciparum* could be detected even in the absence of HRP2 expression. There were no infections with *P. malariae* diagnosed in study participants between 2014 and 2018. We also excluded 5,048 duplicated entries, defined as those with the same patient’s name, patient’s mother’s name and date of malaria testing.

For record linkage, we capitalized all names (individual and mother’s name) and removed accents of accentuated letters and UTF-8 characters that are not *latin-1* (to avoid non-textual characters). We also removed name connectors such as “de” and “dos” (for example, surnames like “dos Santos” became “Santos”). Finally, we replaced common name abbreviations with the full name—e.g., “FCO” and “RDO” were replaced with “Francisco” and “Raimundo”, respectively)—and corrected common name typos. For records with missing date of birth, we assigned January 1 of year obtained from the difference between the year of examination and the age as the date of birth of the patient.

To determine whether a SIVEP-Malaria entry $i$ could be linked to a study participant $j$, we searched the database for case records that matched the patient’s name, the patient’s mother’s name, sex, age and date of birth. We first computed the Jaro-Winkler distance [2], using 0.05 as a prefix scale, to assess the similarity between names. Specifically, we calculated the distances $dₙ(i, j)$ and $dₘ(i, j)$ between the name and mother’s name, respectively, between SIVEP entry $i$ and study participant $j$. A SIVEP-Malaria entry was associated with one or more study participants with the same sex, year of birth (of ±1 year) that satisfied all three conditions below:

1. $dₙ\left( i, j \right)\leq\tau$;
2. $dₘ(i, j)\leq\tau$;

$d\left( i, j \right)=1-\left( 1-dₙ\left( i, j \right) \right)\left( 1-dₘ\left( i, j \right) \right)\leq\tau;$

where $\tau$ is a predefined threshold distance for name similarity (calculated as described in the next section). If the mother’s name of a SIVEP-Malaria entry $i$ or a study participant $j$ was missing, conditions 1-3 were substituted to simply $dₙ\left( i, j \right)<0.1$ [3]. If the participant’s name and mother’s name were both available, conditions 1-3 could also be substituted by identical phonetic representation of name and mother’s name between SIVEP entry $i$ and study patient $j$, as implemented in the package *SoundexBR* [4]. If the SIVEP entry $i$ was associated with multiple study participants, we chose the participant with closest name (i.e., the participant $j$ with minimum $dₙ\left( i, j \right)$).

To define the optimal value of $\tau$, we ran our matching algorithm for a subset of SIVEP-Malaria entries from October 1, 2015 to September 30, 2016. The algorithm returned the most likely match (if any) for every SIVEP-Malaria entry. We manually verified one-by-one whether each of the most likely match is a true correspondence. After testing the true positive rate ($\mathrm{TPR}$) and the true negative rate ($\mathrm{TNR}$) over several threshold values, we generated a receiver operating characteristic (ROC) curve to evaluate the accuracy of the method (Supplementary Figure 3A).

In order to choose the threshold $\tau$, we maximized the reward function

$$R\left( \tau\right)=\mathrm{TPR}\left( \tau\right)+\lambda\cdot\mathrm{TNR}\left( \tau\right),$$

where $\lambda$ is a constant that controls the rate between false positives and false negatives. Supplementary Figure 3B shows $R(\tau)$ as a function of $\tau$ for $\lambda\in\{1,2,\ldots,10\}$ and demonstrates that the optimal threshold $\tau_{opt}$ (which maximizes the function $R(\cdot)$) is relatively stable across different values of $\lambda$. We chose to use $\lambda=5$ to penalize a false positive more than a false negative, resulting in a threshold $\tau_{opt}=0.2668$. To better visualize the effect of $\tau$ on $\mathrm{TPR}$ and $\mathrm{TNR}$, we show in Supplementary Figure 3C that $\mathrm{TNR}$ increases quickly for $\tau>0.3$.

We next applied an interval equal or greater than 28 days between two or more consecutive cases to count the latter episode as a new malaria infection. When different species were detected in samples obtained less than 28 days apart, the participant was considered to have a single episode of mixed-species infection. Finally, we excluded 114 malaria case records that occurred at a time when the participant did not live in the study site. This resulted in 2,369 malaria records among study participants (2,033 due to *P. vivax*, 298 due to *P. falciparum* and 38 mixed-species infections) (Supplementary Figure 2).

**Duffy blood group genotyping**

The genotyping reaction was performed with a final volume of 10.0 μL, comprising 5.0 μL of Genotyping Master Mix (Thermo Fisher), 0.5 μL of the commercial assay (primers and probes from Thermo Fisher), 2.0 μL of genomic DNA at 2.5 ng/μL (total of 5.0 ng in the reaction), and 2.5 μL of DNase-free PCR water. The reactions were carried out in 96-well plates under the following cycling conditions: one cycle of polymerase activation at 95°C for 10 minutes, followed by 50 cycles of denaturation at 95°C for 15 seconds and annealing/extension at 60°C for 1 minute. Amplification and fluorescence detection were performed using the Applied Biosystems ViiA 7 Real-Time PCR System (Thermo Fisher). Results were analyzed using the QuantStudio Real-Time PCR software v1.3 (Thermo Fisher). All reactions contained positive controls and no-template negative controls. Positive controls consisted of well-characterized samples with known *FY* genotypes, previously validated by multiplex qPCR using SYBR Green I and serotyped with the ID-Card FYA/FYB system (DiaMed AG, Cressiersur Morat, Switzerland).

**Statistical analysis**

Because participants were not consistently present throughout the entire follow-up period, and malaria incidence varied over time, we introduced a covariate to represent the individual's time at risk, weighted by the daily malaria incidence rate. These weights were normalized such that individuals present for the entire study period received a value of 1, while those absent during the entire follow-up period received a value of zero. For an individual that entered the study on day $t_{1}$ and left on day $t_{2}$, the adjusted time at risk is given by,

$$\Delta t=\frac{\sum_{t=t_{1}}^{t_{2}} I\left( t \right)}{\sum_{t=1}^{T} I\left( t \right)},$$

where $T$ is the total duration of the study and $I(t)$ is the number of recorded SIVEP-Malaria cases on day $t$.

Next, we fitted a zero-inflated negative binomial (ZINB) model to case count data and included covariates representing *FY* genotype, sex, age group (0–16, 17–40, and >40 years), and wealth index terciles, along with an interaction term between *FY* genotype and age. We also explored three functional forms for incorporating the individual's weighted time at risk: linear, logarithmic, and exponential. Furthermore, we evaluated whether including age in the zero-inflation component improved model performance while accounting for complexity.

The general form of the ZINB model is given by:

$$\Pr\left( Y_{i}=y \right)=\left\{ \begin{aligned} \pi_{i}+\left( 1-\pi_{i} \right)\cdot p_{NB}\left( 0;\mu_{i},k \right), \mathrm{if} y=0 \\ \left( 1-\pi_{i} \right)\cdot p_{NB}\left( y;\mu_{i},k \right), \mathrm{if} y>0 \end{aligned} \right.,$$

where $\pi_{i}$ denotes the probability that individual $i$ belongs to the structural zero class (i.e., not at risk of malaria during the period of study), $\mu_{i}$ is the expected count for individual $i$ if at risk, $k$ is the dispersion parameter of the negative binomial distribution and $p_{NB}(y;\mu_{i},k)$ denotes the probability mass function of a negative binomial distribution with parameters $\mu_{i}$ and $k$ evaluated at the point $y$.

The model components were specified as:

1. Count component (log link):

$$\log\left( \mu_{i} \right)=\beta_{0}+\boldsymbol{\beta}_{1}\cdot{[\boldsymbol{FY}\mathbf{genotype}]}_{i}+\boldsymbol{\beta}_{2}\cdot{[\mathbf{age}]}_{i}+\beta_{3}\cdot{[sex]}_{i}+\boldsymbol{\beta}_{4}\cdot{[\mathbf{wealth index}]}_{i}+$$

$$\beta_{5}f\left( [{weighted time at risk]}_{i} \right)+\boldsymbol{\beta}_{6}\cdot\left( \left[ \boldsymbol{FY}\mathbf{genotype} \right]_{i}\otimes\left[ \mathbf{age} \right]_{i} \right);$$

2. Dispersion component (log link):

$$\log\left( k_{i} \right)=\delta_{0};$$

3. Zero-inflation component (logit link):

$${\mathrm{logit}(\pi}_{i})=\gamma_{0}+\boldsymbol{\gamma}_{1}\cdot\left[ duffy negative \right]_{i}+\boldsymbol{\gamma}_{2}\cdot\left[ \mathbf{age} \right]_{i}+\gamma_{3}g\left( [{weighted time at risk]}_{i} \right).$$

In these equations:

- $\otimes$ and $\cdot$ denote respectively the Kronecker and dot products between two vectors;
- $\beta_{0}$, $\gamma_{0}$ and $\delta_{0}$ are intercepts;
- $\beta_{k},1\leq k\leq5$ and $\gamma_{k},1\leq k\leq3$ are regression coefficients associated with categorical covariates. Bold coefficients represent vectors of regression parameters associated with categorical covariates that have more than two levels (e.g.: $\boldsymbol{\beta}_{2}\boldsymbol{=\{}\beta_{0-16}(ref.),\beta_{17-40},\beta_{>40}\}$)., and bold covariates represent the binary vector representation of categorical covariate;
- $f(\cdot)$ and $g(\cdot)$ denote the functional transformation applied to the weighted time at risk (linear, logarithmic, or exponential); and
- the interaction between *FY* genotype and age is included only in the count component and is represented by the term $\boldsymbol{\beta}_{6}\cdot(\left[ \boldsymbol{FY}\mathbf{genotype} \right]_{i}\otimes{[\mathbf{age}]}_{i})$.

**Model selection**

Using the test-holdout approach, we identified the model offering the best predictive performance, measured by the global deviance in the test set. Candidate models differed in (i) whether the covariate describing individual's time at risk weighted by malaria incidence was introduced in linear, logarithmic, or exponential form; (ii) whether age was incorporated in the zero-inflation component of the model; (iii) whether a binary variable encoding individual's Duffy-positive or Duffy-negative status would replace the genotype variable in the binary compartment; (iv) whether to use the ZINB or Negative Binomial (NB) model. The selected model (i) employed a logarithmic function in both compartments ($f(x)=g(x)=\log(x))$; (ii) Included age in the binary compartment; (iii) employed the binary variable describing the Duffy-positiveness status in the binary compartment; (iv) was selected as a ZINB model. The normality of residuals is a key assumption of our model, we analyzed the normal Q-Q plot and the randomized detrended normal Q-Q plot, or worm plot. Supplementary Figure 5 shows that the residuals align well with a straight line (panel A) and show a random scatter of points around the zero line (panel B), indicating that the residuals follow a normal distribution and supporting the adequacy of the model fit.

**Incidence rate ratios (IRRs), confidence intervals, and *P* values**

*1. Expression of the IRR for the ZINB model*

According to the selected ZINB model, the expected number of infections for an individual in age group $a$ and with *FY* genotype $g$ is

where $\boldsymbol{\beta}_{1},\boldsymbol{\beta}_{2},\boldsymbol{\beta}_{6},\tilde{\boldsymbol{\beta}},\gamma_{1},\boldsymbol{\gamma}_{2},\gamma_{3}$ are model coefficients (as defined in the Statistical analysis subsection), $\boldsymbol{g}$ and $\boldsymbol{a}$ are binary vectors encoding respectively genotype and age, $g^{-}$ is a binary variable that is assigned to 1 if the individual is Duffy-negative (and 0 otherwise), $z$ is the logarithm of the adjusted time of risk, $\sigma\left( t \right)=1/{(1+e^{-t})}$ is the sigmoid function and $\otimes$ is the Kronecker product.

To compute IRRs, we compare individuals with different genotypes but in the same age group and with same $\tilde{\boldsymbol{x}}$. The point estimate for the IRR for two individuals with genotypes $g$ and $g^{'}$ (where $g^{'}=$ $FY^{*}01/FY^{*}01N.01$ is the reference genotype), is

$$\mathrm{IRR}=\frac{\mathbb{E}\left( Y \right)}{\mathbb{E}\left( Y^{'} \right)}=\frac{e^{\boldsymbol{\beta}_{1}\boldsymbol{\cdot g}+\boldsymbol{\beta}_{2}\cdot\boldsymbol{a}+\boldsymbol{\beta}_{6}\cdot\left( \boldsymbol{a}\otimes\boldsymbol{g} \right)+\tilde{\boldsymbol{\beta}}\cdot\tilde{\boldsymbol{x}}}\left( 1-\sigma\left( \gamma_{1}g^{-}+\boldsymbol{\gamma}_{2}\cdot\boldsymbol{a}+\gamma_{3}z \right) \right)}{e^{\boldsymbol{\beta}_{1}\boldsymbol{\cdot}\boldsymbol{g}^{\boldsymbol{'}}+\boldsymbol{\beta}_{2}\cdot\boldsymbol{a}+\boldsymbol{\beta}_{6}\cdot\left( \boldsymbol{a}\otimes\boldsymbol{g}^{\boldsymbol{'}} \right)+\tilde{\boldsymbol{\beta}}\cdot\tilde{\boldsymbol{x}}}\left( 1-\sigma\left( \boldsymbol{\gamma}_{2}\cdot\boldsymbol{a}+\gamma_{3}z \right) \right)}$$

Therefore, if $g$ and $g'$ are Fy-positive genotypes, the IRR of the incidence reduces to the IRR of the count component of the model. Confidence intervals and *P* values for this case can be easily obtained, as explained below. A different approach will be to obtain confidence intervals and *P* values for the Duffy-negative genotype.

*2. IRR for Fy-positive genotypes ($g^{-}=0$)*

To compare different Fy-positive genotypes in the same age group, we computed the estimated effect and corresponding *P* value for linear combinations of model coefficients. For instance, to assess the effect of the interaction between genotype *FY*02/FY*01N.01* and the age category 17-40 years on the count component of the ZINB model, we tested the linear combination:

$$\beta_{FY*02/FY*01N.01}+\beta_{FY*02/FY*01N.01:17-40},$$

where $\beta_{FY*02/FY*01N.01}$ is the effect of *FY*02/FY*01N.01* genotype for age 0-16, and $\beta_{FY*02/FY*01N.01:17-40}$ is the interaction term between *FY*02/FY*01N.01* genotype and the 17-40 age group.

We constructed a vector $\boldsymbol{c}$ with entries equal to 1 for the relevant coefficients in the linear predictor and 0 elsewhere. The estimated effect and its standard error were calculated as, respectively,

$$\hat{\theta}= \boldsymbol{c}^{T}\hat{\beta}\mathrm{and}SE\left( \hat{\theta} \right)=\sqrt{\boldsymbol{c}^{T}\hat{\Sigma}\boldsymbol{c}},$$

where $\hat{\theta}$ is the estimated linear combination of regression coefficients, and $\hat{\Sigma}$ is the estimated covariance matrix of the parameter estimates. We then computed the z-statistic

$$z=\frac{\hat{\theta}}{SE\left( \hat{\theta} \right)}$$

and obtained a two-sided *P* value using the standard normal cumulative distribution function $\phi$ as

$$P=2\cdot\left[ 1-\phi\left( \left| z \right| \right) \right].$$

This approach allows inference on linear combinations of coefficients while fully accounting for the covariance structure of the parameter estimates.

*3. IRR for Fy-negative genotype ($g^{-}=1$)*

The IRR of Fy-negative genotypes and the corresponding confidence intervals and *P* value are calculated using a parametric bootstrap approach. First, we compute point estimates of the IRR, denoted as $\mathrm{IRR}_{\mathrm{point}},$ using Equation (2). Then, $B={10}^{6}$ bootstrap samples of the model coefficients $(\boldsymbol{\beta},\boldsymbol{\gamma},\boldsymbol{\delta})$ are drawn from a multivariate normal distribution with mean given by the point estimate of the model coefficients and covariance matrix given by the estimated covariance matrix $\hat{\Sigma}$.For each parameter sample, we computed an IRR sample and obtained confidence intervals by computing the 2.5% and 97.5% quantiles of the collection of IRR samples. To obtain bootstrap *P* values, we converted each IRR samples $\mathrm{IR}R_{i}$ to a modified IRR (denoted as $\mathrm{IR}R_{null,i}$) assuming the null hypothesis that the expected value of the $\mathrm{IRR}$ is 1:

$$\mathrm{IR}R_{null,i}=\mathrm{IR}R_{i}-\frac{1}{B}\sum_{i=1}^{B} \mathrm{IR}R_{i}+1.$$

The *P* value is evaluated as $P=\frac{M}{B}$, where $M$ is the number of samples satisfying

$$\left| \mathrm{IR}R_{\mathrm{null},i}-1 \right|\geq\left| \mathrm{IRR}_{\mathrm{point}}-1 \right|.$$

*4. IRR marginalized by age or FY genotype*

The bootstrap approach used to estimate IRR for Fy-negative genotypes is also employed to obtain IRR estimates by genotype or age alone (Figure 2A, main text) and corresponding *P* values. We generated IRR bootstrap samples for each genotype $g$ and age group $a$ (denoted as $\mathrm{IR}R_{i}(a,g)$) as described above, and compute the IRR by genotype or age alone as

$$\mathrm{IRR}_{i}\left( g \right)=\sum_{a} w_{A,a}\mathrm{IRR}_{i}(a,g),$$

$$\mathrm{IRR}_{i}\left( a \right)=\sum_{g} w_{G,g}\mathrm{IRR}_{i}(a,g),$$

where $w_{A,a}$ and $w_{G,g}$ are the proportion of the study population respectively in age group $a$ and with genotype $g$.

**Statistical model assumptions**

Our statistical analysis was designed to systematically account for the different components underlying our biological assumptions. The presence of individuals with no or very limited exposure justified the inclusion of a zero-inflated component. By incorporating this component, the regression model distinguishes between zero counts arising from individuals who cannot experience the event of interest and those generated by the usual count process, thereby improving accuracy and reducing bias. Nevertheless, this component alone cannot fully capture population heterogeneity. Rather than assuming that exposed individuals develop malaria purely at random—as implied by a Poisson distribution—we employed a negative binomial model, which assumes that event counts follow a Poisson process with a gamma-distributed rate. Since the observed covariates, including those collected in our study, do not fully capture variance in risk, the negative binomial provides a more appropriate framework for modeling the heterogeneous risk of infection attributable to unobserved factors. Moreover, the model and study design allowed us to assess the statistical significance of the association between age and *FY* genotype, after adjusting for other covariates commonly linked to *P. vivax* incidence.

**Household-level random effects**

Because individuals were clustered within households, a standard approach would be including household-level random effects in the regression models to account for intra-household correlation. However, since the Fy-negative phenotype is associated with African ancestry and individuals frequently cohabit with others of similar ancestry, we evaluated the potential for confounding between household-level effects and *FY* genotype. To investigate this, we first fitted a ZINB model including only the weighted time at risk in both the zero-inflation and count components, along with household-level random effects included in the count component. The household-level random effect estimates were further used to perform a linear regression to examine their association with individual *FY* genotypes (Supplementary Table 3).

Participants carrying the *FY*01N.01/FY*01N.01* genotype had significantly lower household random effect estimates, consistent with the hypothesis that household-level random effects could absorb part of the genotype-related variation. Consequently, to avoid biased inference, we did not include household-level random effects in the final models.

**SUPPLEMENTARY RESULTS**

**Hardy-Weinberg equilibrium**

The frequency of the *FY*01N.01* allele, defined by the -67T→C substitution in the GATA1 transcription factor binding motif (rs2814778), deviated significantly from Hardy-Weinberg expectations, with an excess of wild-type and mutated homozygotes and reduced frequency of heterozygotes (Supplementary Table 2). These findings are consistent with some degree of inbreeding in the study population. By contrast, the frequency of the*FY*01* allele, defined by the 125G→A substitution in the coding region of the *FY* gene (rs12075), followed the Hardy-Weinberg expectation.

**Age as an effect modifier**

Figure 3 (main text) shows that the differences in IRR between the *FY*01/FY*01N.01* and *FY*02/FY*01N.01* genotypes seen in participants aged 0-16 years are attenuated in older age groups. Indeed, all Fy-positives aged >40 years are at similar risk of *P. vivax* infection, regardless of the *FY* genotype.

To further illustrate how age modified the effect of the 125G→A substitution on vivax malaria risk, we show IRR estimates for each age stratum obtained from the NB component of the best-fitting ZINB model using participants aged 0-16 years carrying the *FY*01/FY*01N.01* genotype as the low-risk reference category (Supplementary Figure 6).

**Risk heterogeneity and age**

In our study, the individual-level risk of infection was largely heterogeneous even after adjusting with the ZINB model. To quantify how this heterogeneity varied by age, we computed the probability distributions $f_{i}(x)$ and $g(x;a)$ of the number of infections predicted by the ZINB model for respectively the $i$-th individual and a random individual in age group $a$. For each individual $i$ in age group $a$, we computed the Kullback-Leibler divergence $D_{KL}(f_{i}(x)||g(x;a))=\sum_{x} f_{i}\left( x \right)\log\left( {f_{i}\left( x \right)}/{g\left( x;a \right)} \right)$, a metric that quantifies the distance between $f$ and $g$.

Supplementary Figure 7 shows the distribution of the obtained $D_{KL}$ values across individuals, disaggregated by age and genotype. Among Duffy-positive individuals, there is a clear pattern of decreasing divergence (and therefore reduced heterogeneity) with age, suggesting that the distribution of the expected number of infections become increasingly similar across genotypes in older age groups. This effect supports the hypothesis that accelerated immunity development in younger high-risk individuals results in a convergence of genotype-specific risk in older age groups.

**SUPPLEMENTARY REFERENCES**

1. Vera-Arias CA, Holzschuh A, Oduma CO, Badu K, Abdul-Hakim M, Yukich J, Hetzel MW, Fakih BS, Ali A, Ferreira MU, Ladeia-Andrade S, Sáenz FE, Afrane Y, Zemene E, Yewhalaw D, Kazura JW, Yan G, Koepfli C. High-throughput *Plasmodium falciparum* *hrp2* and *hrp3* gene deletion typing by digital PCR to monitor malaria rapid diagnostic test efficacy. Elife. 2022;11:e72083. doi: 10.7554/eLife.72083.

2. Winkler W. String comparator metrics and enhanced decision rules in the Fellegi-Sunter model of record linkage. American Statistical Association 1990 Proceedings of the Section on Survey Researc Methods, pp. 354–359.

3. Corder RM, de Lima ACP, Khoury DS, Docken SS, Davenport MP, Ferreira MU. Quantifying and preventing Plasmodium vivax recurrences in primaquine-untreated pregnant women: An observational and modeling study in Brazil. PLoS Negl Trop Dis. 2020; 14:e0008526. doi: 10.1371/journal.pntd.0008526

4. Marcelino D. SoundexBR: Soundex (Phonetic) Algorithm for Brazilian Portuguese. R package version 1.2, <http://CRAN.R-project.org/package=SoundexBR>, 2015.**SUPPLEMENTARY FIGURES**


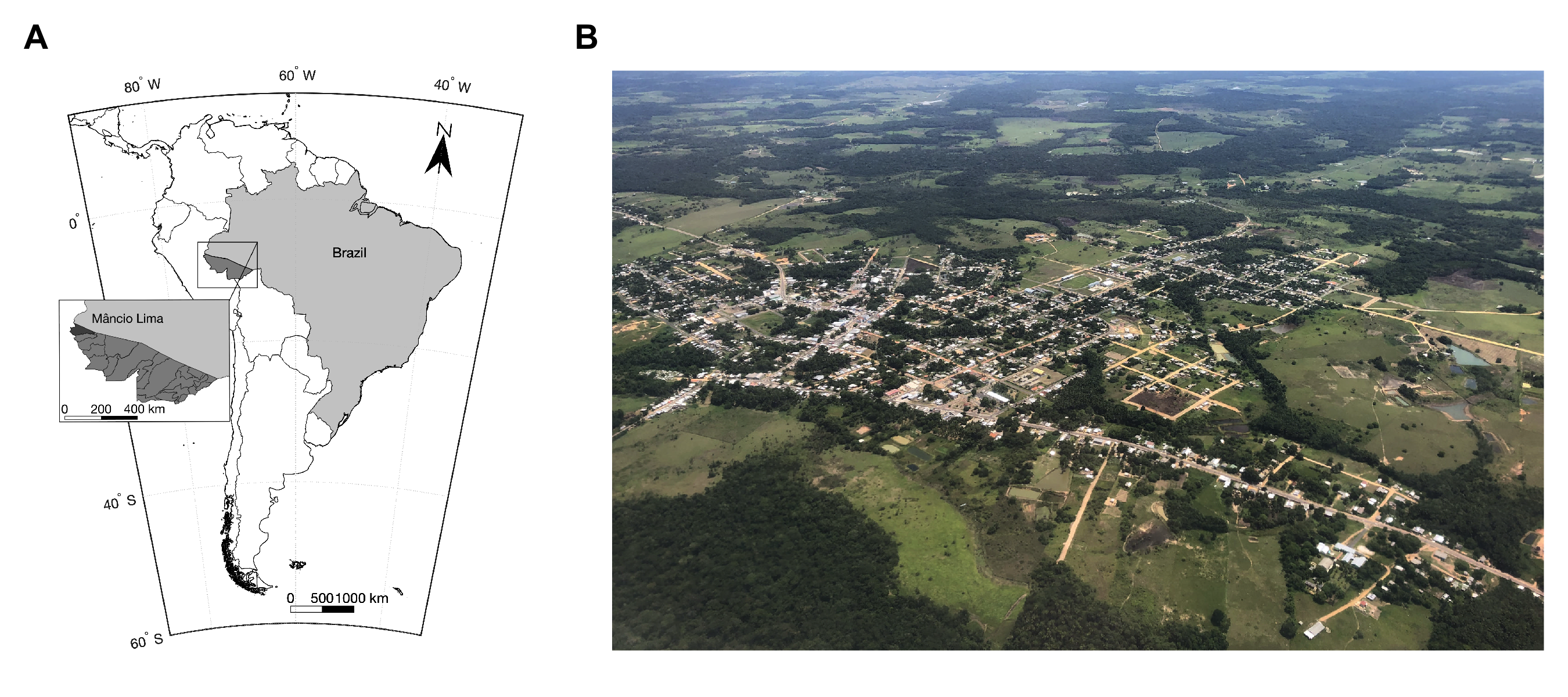


**Supplementary Figure 1.** Study site. (A) Location of the municipality of Mâncio Lima (black) in Acre State (grey) in the western part of Brazil (light grey), next to the border with Peru. (B) Aerial photography of the municipality seat, the town of Mâncio Lima, taken by the senior author.


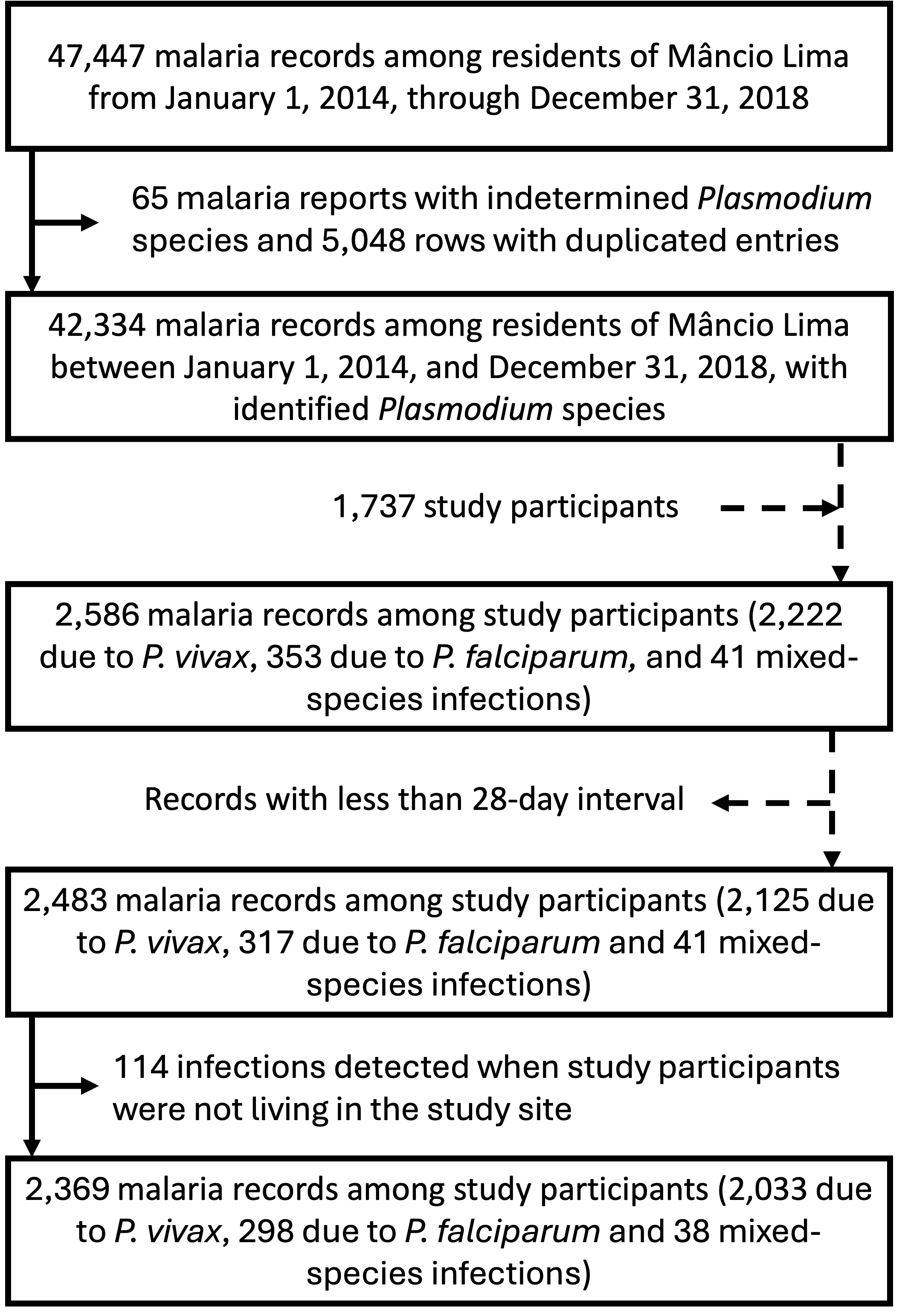


**Supplementary Figure 2.** Study outcome. Malaria case records from 01 January 2014 through 31 December 2018 were retrieved from the SIVEP-Malaria database and matched to study participants as described in Supplementary Methods.

**
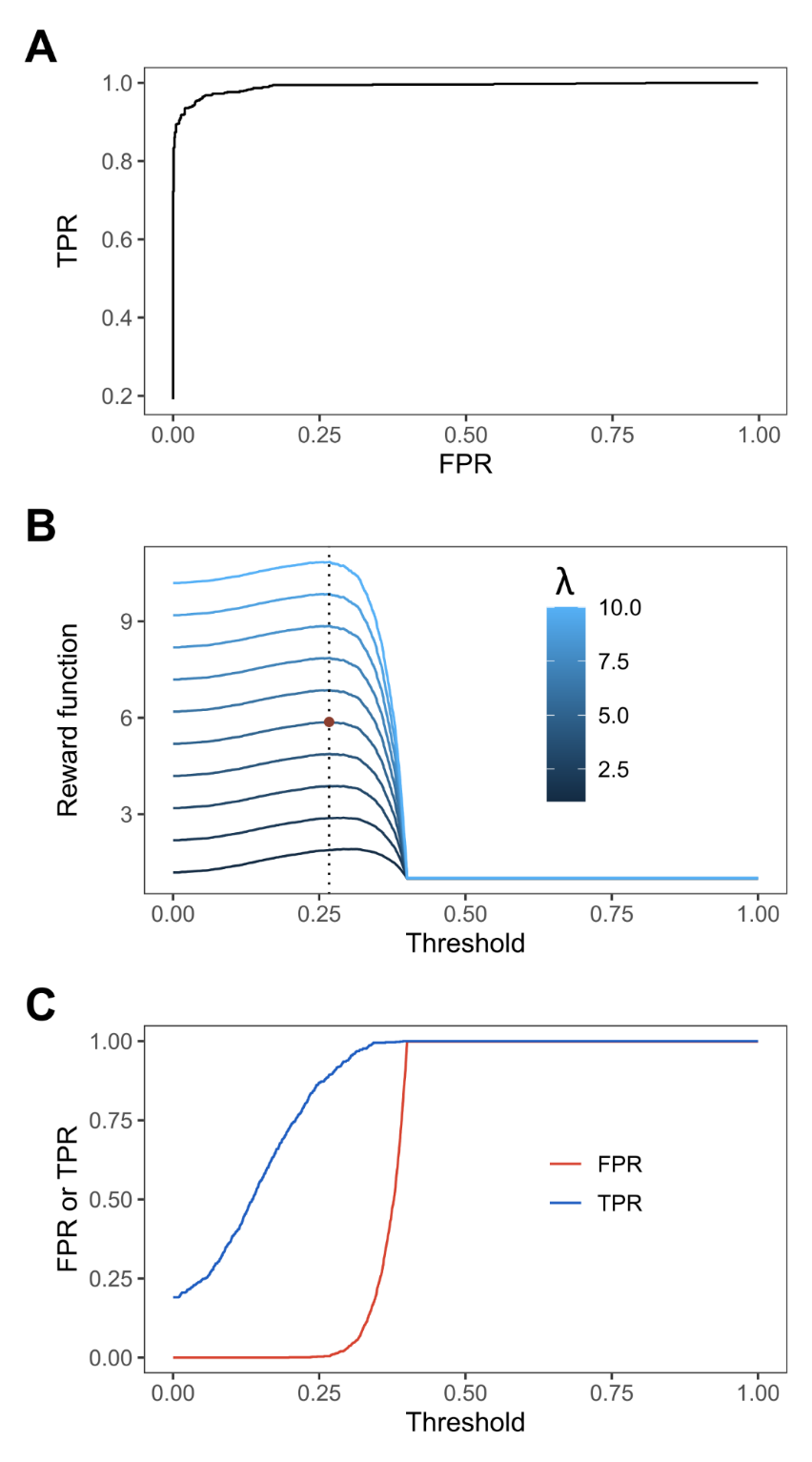
**

**Supplementary Figure 3.** Optimal threshold for record linkage. (A) Receiver operating characteristic (ROC) curve (true positive rate [$\mathrm{TPR}$] *versus* the false positive rate [$\mathrm{FPR}$]) generated by various threshold $\tau$ values. (B) Reward function $R(\tau)$ as function of the threshold $\tau$ for different values of $\lambda\in\{1,2,\ldots,10\}$ (constant that controls the rate between false positives and false negatives) For $\lambda= 5$, the $\tau$ value that maximizes $R(\tau)$ is $\tau_{opt} = 0.2668$. (C) $\mathrm{TPR}$ and $\mathrm{FPR}$ as a function of the threshold $\tau$. For details, see Supplementary Methods.


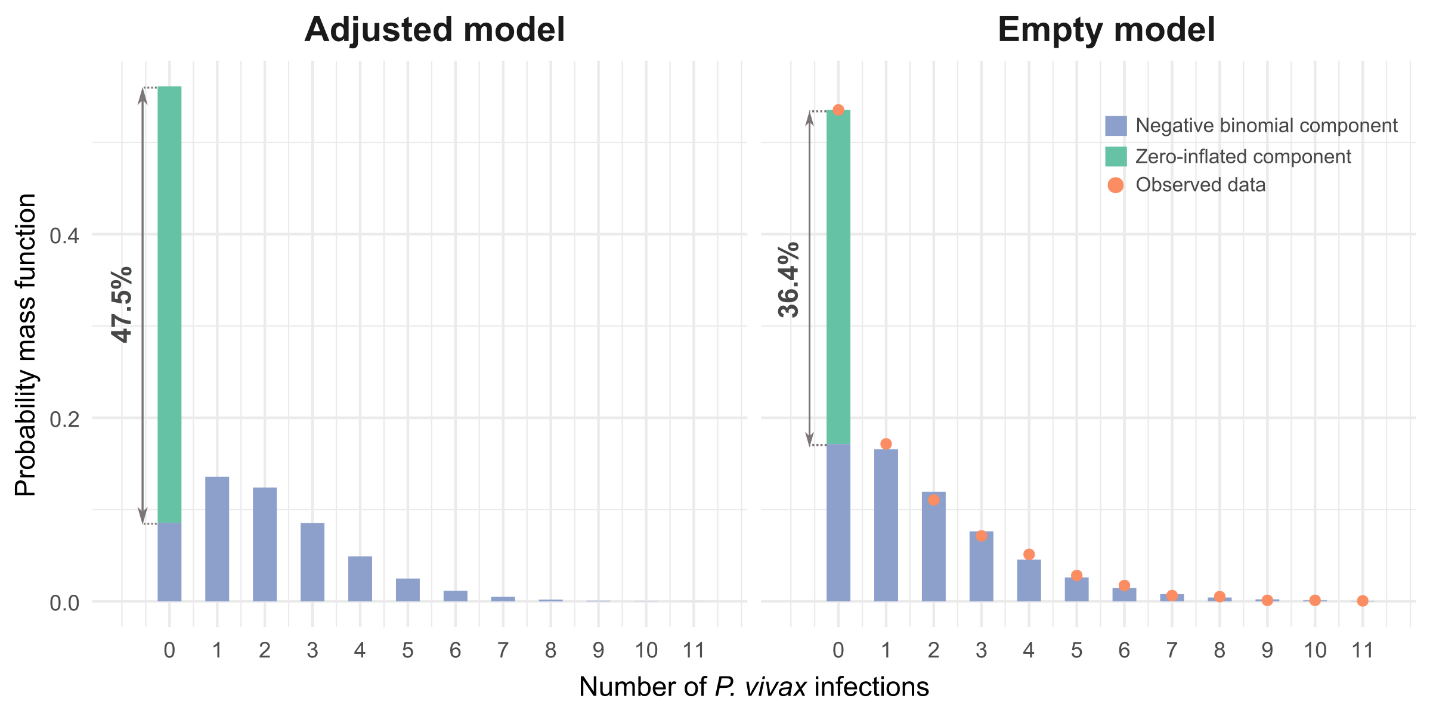


**Supplementary Figure 4.** (A) Distribution of the number of infections per individual predicted by the fully adjusted zero-inflated negative binomial (ZINB) model. (B) Distribution of the number of infections per individual predicted by the empty ZINB model, with no covariates incorporated (bars), fitted to vivax malaria episode counts per person (orange dots) over five years of follow-up in Mâncio Lima, Brazil, 2014-18. The blue bars indicate the number of *P. vivax* infection episodes per person as estimated by the negative binomial function, while the green bar segment indicates the not-at-risk fraction of the population, as estimated by the binary component of the ZINB model.


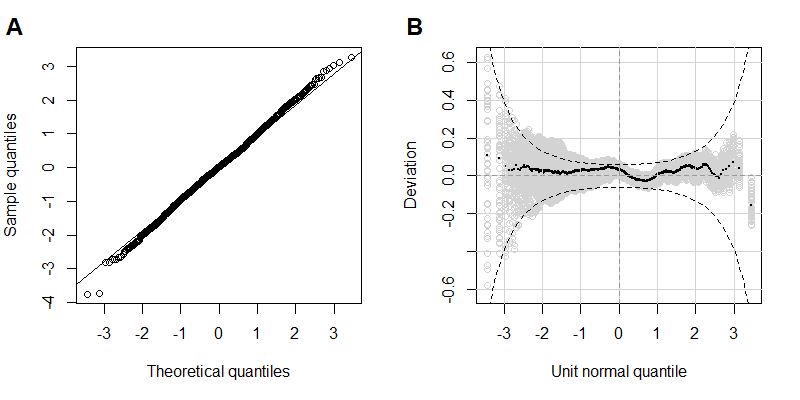


**Supplementary Figure 5.** Model diagnostic plots. (A) Normal quartile-quartile (Q-Q) plot shows a close agreement between the sample and theoretical quantiles, indicating that the residuals are approximately normally distributed and the model fit is adequate. (B) Randomized detrended normal Q-Q plots (worm plots) displays 50 randomized detrended Q-Q plots (gray points) along with their average (black points). The vast majority of the average points fall within the 95% confidence intervals of the expected deviation, further supporting the adequacy of the model fit.
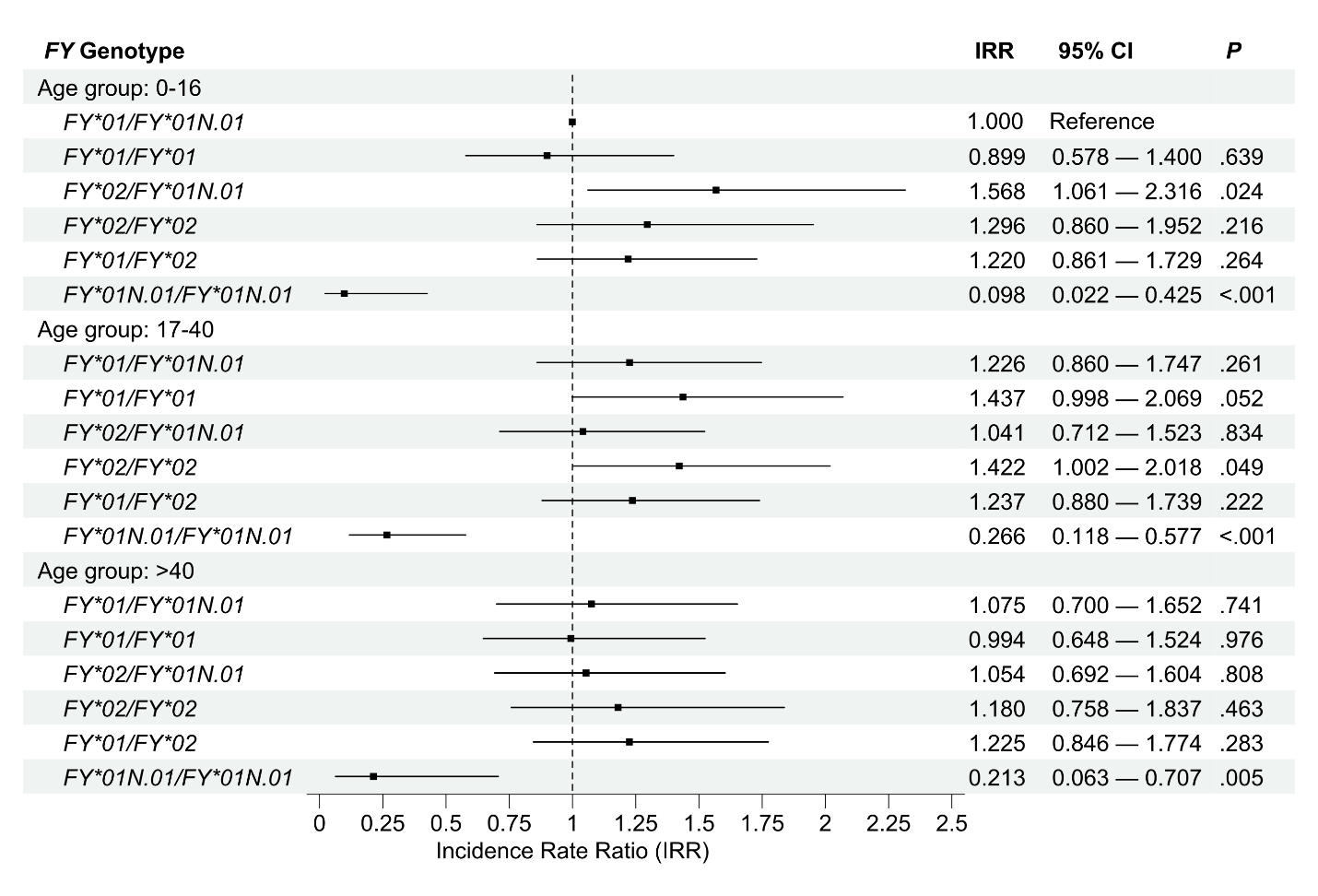


**Supplementary Figure 6.** Incidence rate ratios of vivax malaria risk according to *FY* genotype and age, using participants aged 0-16 years carrying the *FY*01/FY*01N.01* genotype as the low-risk reference category.


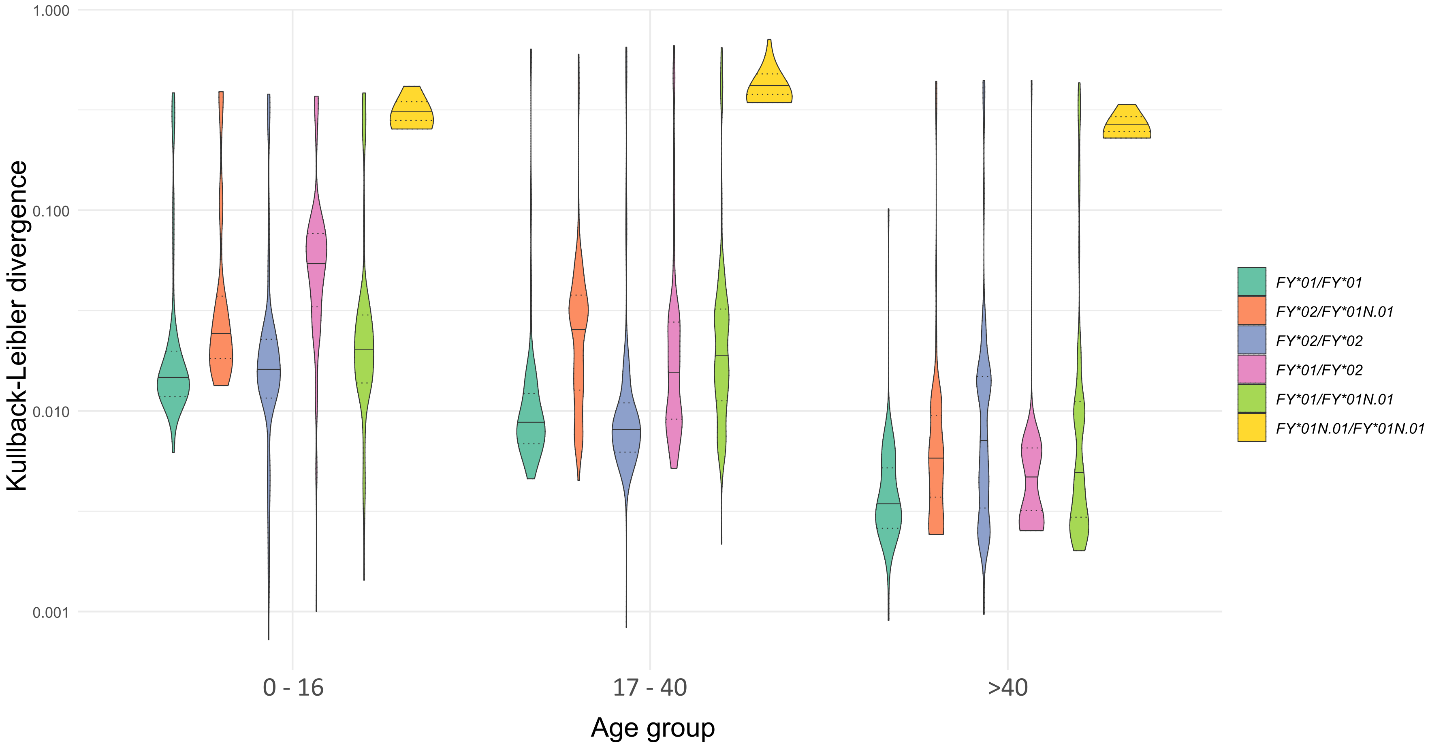


**Supplementary Figure 7.** Violin plots representing the individual-level variation of the Kullback-Leibler divergence between the distribution of the predicted number of cases for a given individual and for a random individual in the same age group. Lower divergence values indicate greater risk homogeneity among individuals in the same group.

**Supplementary Table 1.** Microscopy-confirmed *Plasmodium vivax* malaria episodes recorded in Fy-negative study participants during the follow-up, Mâncio Lima, Brazil, 2014-18.

| **Patient #** | **Sex** | **Age (years)** | **No. of episodes** | **Follow-up duration (days)** |
| --- | --- | --- | --- | --- |
| 1 | M | 3 | 1 | 1826 |
| 2 | M | 12 | 1 | 1826 |
| 3 | F | 15 | 1 | 1828 |
| 4 | M | 15 | 1 | 1826 |
| 5 | M | 19 | 1 | 946 |
| 6 | F | 19 | 1 | 1826 |
| 7 | F | 24 | 1 | 1826 |
| 8 | M | 25 | 2 | 1826 |
| 9 | F | 27 | 1 | 834 |
| 10 | M | 32 | 2 | 1826 |
| 11 | M | 34 | 1 | 1826 |
| 12 | F | 35 | 1 | 1826 |
| 13 | F | 39 | 2 | 1826 |
| 14 | F | 49 | 2 | 1826 |
| 15 | F | 49 | 1 | 1826 |

M = Male, F = Female.

**Supplementary Table 2.** Testing for Hardy-Weinberg equilibrium at the *FY* locus.

| **-67T→C substitution (rs2814778)** | | |
| --- | --- | --- |
| **Genotype** | **Expected frequency** | **Observed frequency** |
| Wild type, homozygous | 1047.67 | 1067 |
| Heterozygous | 602.66 | 564 |
| *FY*01N.01*, homozygous | 86.67 | 106 |
| Allele frequency p: 0.7766 | Chi-squared value: 7.1486 | |
| Allele frequency q: 0.2234 | *P* = 0.0075 | |
| **125G→A substitution (rs12075)** | | |
| **Genotype** | **Expected frequency** | **Observed frequency** |
| *FY*02,* homozygous | 669.02 | 670 |
| Heterozygous | 817.96 | 816 |
| *FY*01,* homozygous | 250.02 | 251 |
| Allele frequency p: 0.6206 | Chi-squared value: 0.0100 | |
| Allele frequency q: 0.3794 | *P* = 0.9203 | |

**Supplementary Table 3.** Household random-effect predictors of the zero-inflated negative binomial (ZINB) model and their association with *FY* genotypes.

| ***FY* genotype** | **Estimate** | ***P*** |
| --- | --- | --- |
| *FY*01/FY*01N.01* | 1.00 | - |
| *FY*01/FY*01* | 1.07 | 0.168 |
| *FY*02/ FY*01N.01* | 1.03 | 0.582 |
| *FY*02/FY*02* | 1.11 | 0.040 |
| *FY*01/FY*02* | 1.10 | 0.035 |
| *FY*01N.01/FY*01N.01* | 0.74 | <0.001 |
